# Development of Multicolor Flow Cytometry Calibration Standards: Assignment of Equivalent Reference Fluorophores (ERF) Unit

**DOI:** 10.6028/jres.116.012

**Published:** 2011-06-01

**Authors:** Lili Wang, Adolfas K. Gaigalas

**Affiliations:** National Institute of Standards and Technology, Gaithersburg, MD 20899

**Keywords:** allophycocyanin (APC), equivalent reference fluorophores (ERF), fluorescein isothiocyanate (FITC), fluorescence, multicolor flow cytometer, Pacific Blue (PB), phycoerythrin (PE)

## Abstract

A procedure is described for assigning the number of equivalent reference fluorophores (ERF) values to microspheres labeled with a fluorophore designed to produce a fluorescence response in a given fluorescence channel of a multicolor flow cytometer. A fluorimeter was calibrated by a series of solutions of the reference fluorophores. The fluorimeter was used to obtain the microsphere fluorescence intensity, and a multicolor flow cytometer was used to obtain the microsphere concentration. The microsphere fluorescence intensity and the concentration were used to obtain the value of ERF for each model microsphere calibration standard. The procedure is described in detail only for microspheres with allophycocyanin (APC) immobilized on the surface. ERF values were also determined for microsphere calibrators for three other fluorescence channels: fluorescein isothiocyanate (FITC), phycoerythrin (PE), and Pacific Blue(PB). The four model microsphere calibrators provide a one point calibration for the four channels of a flow cytometer. By using software controls and changing the photomultiplier voltages, it is possible to obtain a multipoint calibration for each fluorescence channel using each microsphere calibrator.

## 1. Introduction

Multicolor flow cytometers are used extensively in research and clinical settings [1–3]. Cells are incubated with labeled antibodies specific to antigens on the surface or inside of a cell. Antibodies with spectrally different labels are detected in different fluorescence channels of the flow cytometer and are indicative of the presence of specific surface or intracellular antigens that are the targets of the labeled antibodies. The objective of quantitative flow cytometry is to relate the fluorescence intensities in a given fluorescence channel to the number of the labeled bound antibodies on the cell surface or within the cell [4, 5]. Each fluorescence channel should give an estimate of the number of bound antibodies labeled with the fluorophore assigned to that channel. In a previous publication, we proposed a method to accomplish this objective in two steps [6].

The first step is the establishment of a linear fluorescence intensity scale for each fluorescence channel. The second step is to convert the intensity scale to a scale giving the number of labeled antibodies on the cell. This manuscript describes a procedure to establish a linear scale in four fluorescence channels (fluorescein isothiocyanate (FITC), phycoerythrin (PE), allophycocyanin (APC), and Pacific Blue (PB)) of a multi-color flow cytometer. The simplest possible procedure is a single point calibration of each fluorescence channels. Four CompBeads^1^ populations from BD Biosciences were chosen as the model microsphere calibrators. These microspheres were provided with a coat of rat anti-mouse kappa light chain antibodies, and were incubated with four mouse antibodies labeled with either FITC, PE, APC, or PB fluorophores. After incubation, the number of Equivalent Reference Fluorophores (ERF) was assigned to each microsphere calibrator. The linear scale for a flow cytometer was established by passing the labeled microspheres through a flow cytometer and plotting the measured fluorescence intensity in each channel versus the assigned ERF value of the calibrator. The flow cytometer calibration can be extended to multiple points by changing the photomultiplier voltage. Alternately, a multipoint calibration can also be obtained by using several populations of microspheres each with a different ERF value as shown in a previous work with Ultra Rainbow beads [6]. The development of a standard to translate the linear scale into a scale related to the number of bound antibodies will be described in a subsequent paper.

Considering that most commercially available microsphere calibrators are small in size (2–4 μm) and contain multiple microsphere populations similar to the CompBeads, we have developed this procedure to accurately assign the ERF value to the major microsphere population within the calibrator. The development of clinical multicolor flow cytometer assays is currently a major undertaking. Future multicolor flow cytometer assays are likely to incorporate internal calibration standards so that comparable results can be obtained with any multicolor flow cytometer. The wide-spread implementation of this procedure will ensure the comparability of clinical assay results.

## 2. General Procedure for Assigning ERF Values

**Fig. 1:**
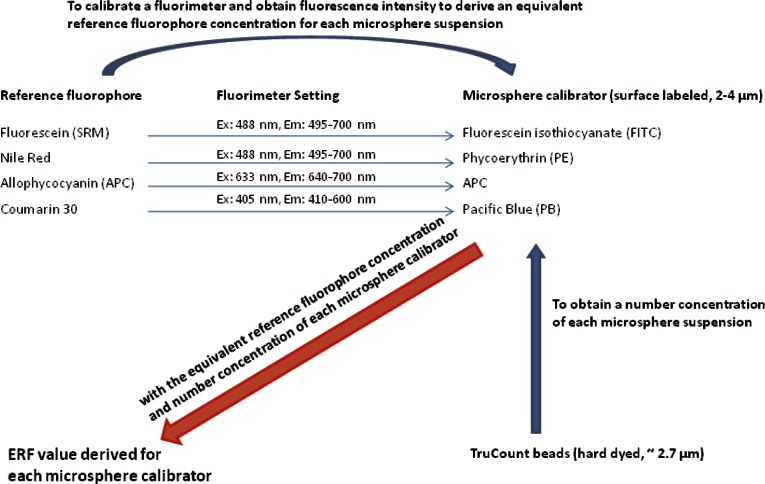
A schematic description of the procedure used to obtain ERF values of four microsphere calibrators using four different reference fluorophore solutions. A value of equivalent concentration of a reference fluorophores is obtained for each microsphere suspension using a fluorimeter calibrated with a set of solutions of a reference fluorophore under a specified instrument setting. The solutions were obtained by serial dilutions of a stock solution. The number concentration of each microsphere suspension was measured using a multicolor flow cytometer and an internal bead counting standard. The two values, the equivalent reference fluorophore concentration and the number microsphere concentration, were used to assign a ERF value to each microsphere calibrator.

The ERF value of a microsphere calibrator is defined as the number of equivalent reference fluorophores which give the same fluorescence intensity as that of a single microsphere calibrator [6]. A summary of the scheme for assigning ERF values to the four microsphere calibrators is given in Fig. 1. First a fluorimeter was calibrated with a set of progressively diluted solutions of a stock solution of each reference fluorophore shown in the left column in Fig. 1. The fluorimeter calibrations related the fluorescence intensity from the solution of reference fluorophores to the concentration of the reference fluorophores. Since the dilution factor was between 5 and 10, the concentrations of the reference solutions tended to group in the lower portion of the axis when plotted on a linear scale. Therefore, several methods of fitting the data were investigated in order to obtain the most representative calibration line. Next, the fluorescence intensity of the microsphere calibrator suspensions (shown in the right column of Fig. 1) was measured. The fluorimeter calibration line was used to relate the fluorescence intensity of the microsphere suspension to the concentration of the corresponding reference fluorophores (shown by the horizontal arrows in Fig. 1). Finally the number concentration of each microsphere suspension was measured using a multicolor flow cytometer and an internal bead counting standard. The two values, the equivalent reference fluorophore concentration and the number microsphere concentration, enabled the assignment of the ERF value to each microsphere calibrator. The following contains an outline of four different fitting procedures used to generate the fluorimeter calibration line, the procedure to find the microsphere concentration, and finally how this data is used to estimate the ERF values of the calibration microspheres.

### 2.1 Fitting Procedures for Obtaining a Calibration Line From a Set of Reference Solutions

Let *x* be a set of concentrations and *y* a set of corresponding fluorescence intensities. A calibration line is obtained by a best linear fit to the pairs of data points denoted by (*x*, *y*). A linear fit assumes that the data points are related by Eq. (1)

(1) $$y=y_{0}+m\cdot x$$

where *m* is the slope and *y*_0_ is the *y*-intercept. If the data is such that the majority of points are located at low values of *x*, then taking a log of the data results in a more uniform distribution of points along the fluorescence intensity and concentration axes. Such a distribution gives each region a similar importance in the fitting. The fitting of the transformed data has to be performed using a more general nonlinear procedure. Performing a log transforming of Eq. (1) leads to

(2) $$\begin{array}{l} \log(y)=\log(mx(1+y_{0}/mx))\\ \;=\log(m)+\log(x)+\log(1+y_{0}/mx)\\ \;=\log(m)+\log(x)+\log(1+y_{0}/m\cdot 10^{\log(x)}). \end{array}$$

Equation (2) suggests that the fitting of the transformed data should use a parameterization given by Eq. (3)

(3) $$\log(y)=a+\log(x)+\log(1+b/10^{\log(x)}).$$

The fit parameters of the transformed data give the slope and *y*-intercept according to the relations:

(4) $$\begin{array}{l} m=10^{a}\\ y_{0}=10^{a}*b. \end{array}$$

Given noiseless data, using either Eq. (1) or Eq. (3) leads to the same result for the slope and intercept. Given data with noise (uncertainties in the values of concentration and fluorescence intensity), the transformed data is more uniformly distributed along the geometric axis, thus the fit is not biased toward any specific region of data.

In practice, the log transformed data is often modeled with a linear relation.

(5) log(y)=a+d⋅log(x).

Equation (5) is a valid representation of the data if the intercept *y*_0_ can be neglected. In that case, the parameter *d* should be equal to 1 and the parameter *a* gives the slope as before, *m* = 10^a^. However if the intercept can not be neglected, then Eq. (5) is equivalent to representing the actual data with a model given by Eq. (6).

(6) $$y=m\cdot x^{d}.$$

The model tries to minimize the effect of neglecting *y*_0_ by introducing a slight curvature in the representation of the data, hence the presence of the parameter *d* in Eq. (6).

### 2.2 Calibration of the Fluorimeter and the Estimate of Equivalent Fluorophore Concentration

The fluorimeter was calibrated using solutions of a reference fluorophore. The dependence of the reference concentration, *C*_ref_, on the fluorescence intensity, *FI*_ref_, was represented by a calibration line given by

(7) $$C_{ref}=C_{0}+m\cdot FI_{ref}.$$

The measured fluorescence intensity from a suspension of microspheres, *FI*_microspheres_, was put into Eq. (7) to define an equivalent concentration of reference solution, *C*_equivalent_, which gives the same fluorescence intensity as the microsphere suspension. As suggested by the previous discussion, there are several ways to estimate the parameters in Eq. (7). The first method is simply to fit the reference data using a linear model. Then *C*_equivalent_ can be evaluated using Eq. (1)

(8) $$C_{equivalent}=C_{0}+m\cdot FI_{microsphere}.$$

In the case of a linear fit to the log transformed reference data, Eq. (5) gives:

(9) $$C_{equivalent}=10^{\left(a+d\cdot \log(FI_{microsphere})\right)}.$$

In the case of a nonlinear fit to the log transformed reference data, Eq. (3) gives:

(10) $$C_{equivalent}=10^{\left(a+\log(FI_{microsphere})+\log(1+\frac{b}{FI_{microsphere}})\right)}.$$

In many situations, it may be possible to neglect the intercept and estimate the slope using one calibration point nearest to the measured microsphere fluorescence intensity. In that case, the equivalent concentration is given by

(11) $$C_{equivalent}=\frac{C_{ref}}{FI_{ref}}FI_{microsphere}.$$

The subscript *ref* in Eq. (11) refers to the calibration point which is nearest to the measured microsphere fluorescence intensity when approached from a larger value on the axis.

### 2.3 Obtaining Microsphere Concentration From Flow Cytometer Measurements

The concentration of microspheres was obtained using the ARIA II flow cytometer and the TruCount beads with a diameter of approximately 2.7 μm (BD Bioscience, San Jose, CA) as an internal counting standard. The hard dyed, single population TruCount beads are packaged as a dry pellet in a tube. The beads give a fluorescence signal in multiple fluorescence channels of the flow cytometer. The number of beads per tube, *N*_TruCount_ is given by the manufacturer.

The test microspheres with the unknown concentration and the counting beads were suspended in a buffer of known volume (e.g., 0.5 mL). For simplicity, the entire content of the tube of the counting beads was suspended in the buffer containing the test suspension. The mixture of the counting beads and test microspheres was passed through the flow cytometer. It was assumed that the relative number of the test microspheres to the counting beads per analytical volume measured by flow cytometer was the same as the relative total number of test microspheres to TruCount beads in the original mixture. Based on this assumption, the working equation is given by Eq. (12)

(12) $$\frac{N_{microsphere}}{N_{TruCount}}=\frac{COUNT_{microsphere}}{COUNT_{TruCount}}$$

where *N*_microspheres_ is the number of test microspheres in the mixture, *COUNT*_microspheres_ is the number of test microspheres measured by the flow cytometer, and *COUNT*_TruCount_ is the number of the counting beads measured by the flow cytometer. Solving Eq. (12) for *N*_microspheres_ and dividing by the volume of the suspension gives the microsphere number concentration as shown in Eq. (13)

(13) $$C_{microspheres}=\frac{COUNT_{microsphere}}{COUNT_{TruCount}}\cdot \frac{N_{TruCount}}{V_{mixture}}.$$

To use Eq. (13) properly it was necessary to identify the test microspheres and the counting beads in the flow cytometric measurement. Usually it is possible to use either scattering or fluorescence signals to distinguish between the two populations of particles. A gate (to be described as P8 in Fig. 5a) is set in the side scatter-forward scatter (SSC-FSC) plot of the total particle events. Counting the events in this gate gives the number of total particles detected, *COUNT*_microspheres_ + *COUNT*_TruCount_. Next, a gate (to be described as P9 in Fig. 5c) is set on the **fluorescence** peak in the histogram associated with the counting beads’ fluorescence in the APC channel. The count in this peak gives the total number of the counting beads detected, *COUNT*_TruCount_. Thus the ratio of the counts is given by Eq. (14)

(14) $$\frac{COUNT_{microsphere}}{COUNT_{TruCount}}=\frac{P8-P9}{P9}$$

where P8 and P9 stand for the total number of events in the corresponding gates. Thus the final concentration of the microspheres is given by Eq. (15)

(15) $$C_{microsphere}=\frac{P8-P9}{P9}\cdot \frac{N_{TruCount}}{V_{mixture}},mL^{-1}.$$

For the case where the TruCount microspheres do not have a fluorescence signal, the procedure given by Eq. (15) has to be modified. In this case, it is necessary to measure a sample of the test microspheres (without the counting beads) with the same concentration of test microspheres as in the mixture of both test microspheres and TruCount beads. Let *P*3*_W_* be a gate around the region in the SSC-FSC scatter plot which contains both the TruCount beads and unknown microspheres. Let be *P*3*_WO_* the same gate for the case of the sample with test microspheres, but without the TruCount beads. Then, Eq. (14) is modified according to Eq. (16)

(16) $$\frac{COUNT_{microsphere}}{COUNT_{TruCount}}=\frac{P8-(P3_{W}-P3_{WO})}{P3_{W}-P3_{WO}}.$$

### 2.4. Assigning ERF Values to Microspheres

The equivalent number of reference fluorophores per microsphere (ERF) is estimated by dividing *C*_equivalent_ with the concentration of microspheres in the suspension, *C*_microspheres_. **Explicitly**

(17) $$ERF=\frac{6.0221*10^{23}}{1000}\frac{C_{equivalent}}{C_{microsphere}}.$$

The constant in Eq. (17) converts the reference concentration from mol/L to mL^−1^.

Inserting one of the estimates of *C*_equivalent_, the result from each of the different models defined by Eqs. (8, 9, 10, or 11) gives an estimate of the ERF value for that microsphere.

Equation (17) gives the measured value of ERF for the microsphere suspension, which may have several distinct populations, each with the same fluorophore, but characterized by a different size and/or fluorophore loading. The different populations are associated with identifiable clusters in the SSC-FSC scatter plot. Most flow cytometer analyses are performed using a single, dominant population. In the case of the APC microsphere calibrator, there are two distinct populations of microspheres (to be described as P1 and P2 gates in Fig. 6a). Let *I*_major_ represent the average fluorescence signal of a microsphere in the major microsphere calibrator population (as seen in P1 gate). The ERF for the major population can be written in terms of the ERF for the total suspension by Eq. (18)

(18) $$ERF_{major}=ERF\cdot (I_{major}/I_{avr})$$

where *I*_avr_ is the average fluorescence response of all microspheres. Equation (18) is justified by noting that the ratio of ERF values describes the relative fluorescence response of individual microspheres and is therefore identical to the ratio of the fluorescence intensities measured in the flow cytometer. Thus, the ratio of fluorescence intensities of two distinct microsphere populations should be identical to the ratio of their respective ERF values. In order to obtain a value for *ERF*_major_ (this is the population which is used in interpreting a flow cytometer measurement), it is necessary to evaluate the fluorescence intensity of the major microsphere population relative to the total microsphere population. This can be performed on a flow cytometer, where forward and side scattering can be used to identify the microspheres in the major and total populations, and the fluorescence signal can measure the fluorescence signal can measure the relative fluorescence intensity of the two populations (to be described as Fig. 6.

## 3. Results

The procedures outlined in the previous section were applied to four microsphere preparations each with a different fluorophore. Detailed results will be given for the APC microsphere calibrator; the results for the other microspheres will be different in values, but the identical procedure was used to obtain them.

### 3.1 Assignment of ERF Value to APC Microsphere Calibrator

The assignment of ERF values to APC microsphere calibrator used reference solutions of APC. The concentration of a stock solution of APC was obtained using amino acid analysis as described previously [6], and its value was (3.76 ± 0.33) mg/mL. Using a molar mass of 106,610 g/mol, the molar concentration of the APC stock solution was estimated to be 35.3 μmol/L [7, 8]. Next, a series of dilutions of the stock solution was performed to obtain a set of solutions with progressively smaller concentrations of APC. Table 1 gives the results of weighing of the APC stock solution to make the first dilution. First a small tube was weighed (column 1 in Table 1), followed by the weighing of the tube containing the APC stock solution (column 2 in Table 1) and finally the tube containing the APC stock solution and PBS buffer, 0.02 % (w/w) Tween 20 (column 3 in Table 1). Each weighing was followed by a measure of the empty balance to determine drifts in the “zero” values, and these drifts were subtracted from the weighings.

The concentration of the new solution, called Sol1, was determined using Eq. (19)

(19) $$C_{Sol1}=C_{APC}\cdot \frac{g_{APC}}{g_{APC}+g_{PBS}}=C_{APC}\cdot dil.$$

The quantities *g*_APC_ and *g*_PBS_ were found from the average of the differences between columns 2 and 1 and columns 3 and 2, respectively. In all cases, the background value was subtracted. The symbol *dil* represents a dilution factor and is defined by Eq. (19) as a ratio of the indicated masses. Equation (19) assumes that the densities of the APC stock solution and the buffer are identical. This assumption introduces systematic errors, which are insignificant compared to the measured uncertainty of the concentration of the APC stock solution. The uncertainty in the concentration of the new solution was found using the standard error propagation expression given in Eq. (20)

(20) $$\sigma_{Sol1}=C_{Sol1}\cdot {\left[{\left(\frac{\sigma_{APC}}{C_{APC}}\right)}^{2}+{\left(\frac{\sigma_{dil}}{dil}\right)}^{2}\right]}^{1/2}.$$

The uncertainty in the *dil* factor was found using the definition in Eq. (19) and an expression similar to Eq. (20). The procedure outlined above for the first dilution of the stock solution of APC was followed to produce additional 5 serial dilutions. The results are shown in Table 2 with the lowest concentration (Solution 6) in the first row. The third column in Table 2 gives the uncertainties, which include the uncertainty of the stock solution of APC and the subsequent uncertainty introduced by the weighing.

### 3.2 Fluorescence Intensities of the Reference Solutions of APC

**Fig. 2:**
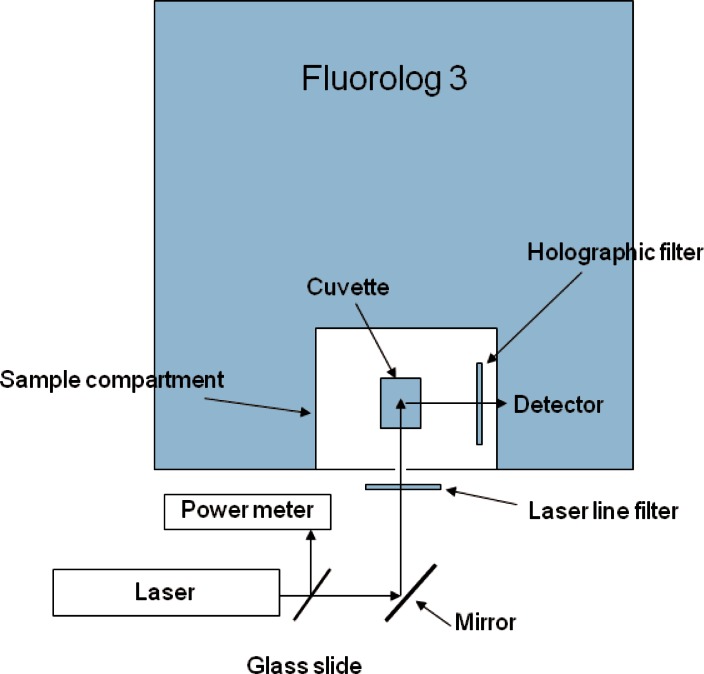
A schematic of the commercial fluorimeter with an external laser, used to illuminate a sample located in the sample compartment. The laser entered the sample compartment through a small hole. A holographic notch filter was placed in front of the detector aperture in the sample compartment. The data was collected using the software associated with the spectrometer.

**Fig. 3:**
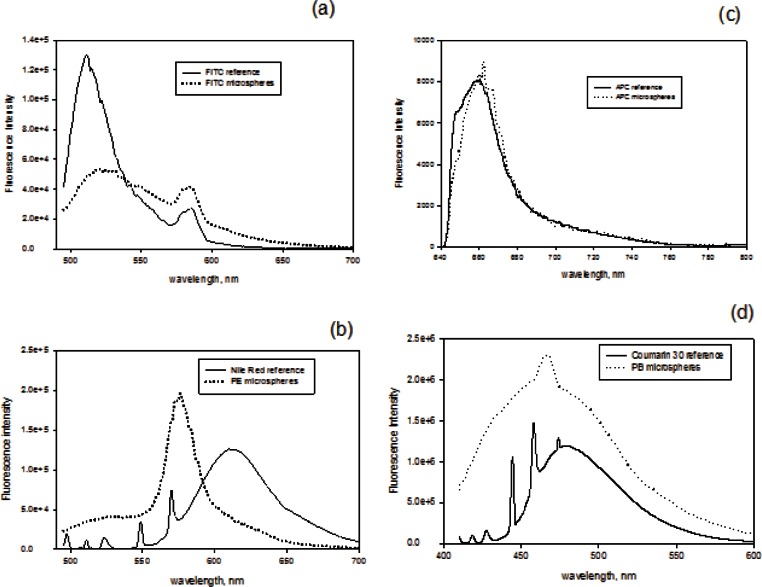
Unsubtracted and uncorrected fluorescence spectra of reference solutions (solid traces) and the associated microsphere calibrators in PBS buffer, pH 7.4, 0.5 % (w/w) BSA and 0.1 % (w/w) sodium azide (dotted trace). (a) FITC in PBS buffer, pH 7.4 and FITC microsphere calibrator with 488 nm laser excitation. (b) Nile Red fluorophores in acetonitrile (ACN) solvent and PE microsphere calibrator with 488 nm laser excitation. (c) APC in PBS buffer, pH 7.4, 0.02% (w/w) Tween 20 and APC microsphere calibrator under a 633 nm laser excitation. (d) Coumarin 30 fluorophores in ACN solvent, and PB microsphere calibrator with 405 nm laser excitation.

The fluorescence was measured using a JY Horiba Fluorolog 3 commercial fluorescence spectrometer which was modified to allow excitation with an external laser. Figure 2 shows the schematic of the illumination arrangement. An external laser beam (a 405 nm laser for PB calibrator, a 488 nm laser for FITC and PE calibrators and a 633 nm laser for APC calibrator) entered the Fluorolog 3 sample chamber through a small aperture in the wall of the sample compartment. A laser line filter was placed in front of the aperture to minimize extraneous light. A holographic notch filter was placed in front of the detector port inside the sample compartment. Measurements were performed by scanning the detector wavelength over the emission region in steps of 1 nm. The detector slit width was set to 2 nm, the integration time was set to 1 s., and the photomultiplier voltage was set to 1000 v. The solid traces in Fig. 3 show the uncorrected fluorescence emission spectrum from reference solutions prior to subtraction of the medium spectrum. The dashed traces in Fig. 3 show the uncorrected spectrum from microspheres assigned to FITC fluorescence channel (a), PE fluorescence channel (b), APC fluorescence channel (c), and Pacific Blue fluorescence channel (d). Prior to analysis, the medium spectra were subtracted from all fluorescence spectra. The subtracted spectra were further corrected for spectral variation of the detector response. The fluorescence intensity was estimated by summing the emission spectrum from the lowest to the highest observed wavelength. In the case of APC, the subtracted and corrected spectrum was summed from 640 nm to 800 nm (Fig. 3c). The fourth column of Table 3 shows the result of the fluorescence measurements for four dilutions of APC stock solution. The concentration data are reproduced from Table 2. Table 3 constitutes the data used for calibration of the fluorescence spectrometer. Similar procedures were followed for the calibration of the fluorimeter with 405 nm and 488 nm excitation (data not shown). The laser line filter and holographic filter were changed to accommodate the excitation laser. Different reference fluorophore solutions were used for calibrating the fluorimeter at the different excitation wavelengths as described in Fig. 1 and in the caption of Fig. 3 [6].

**Fig. 4:**
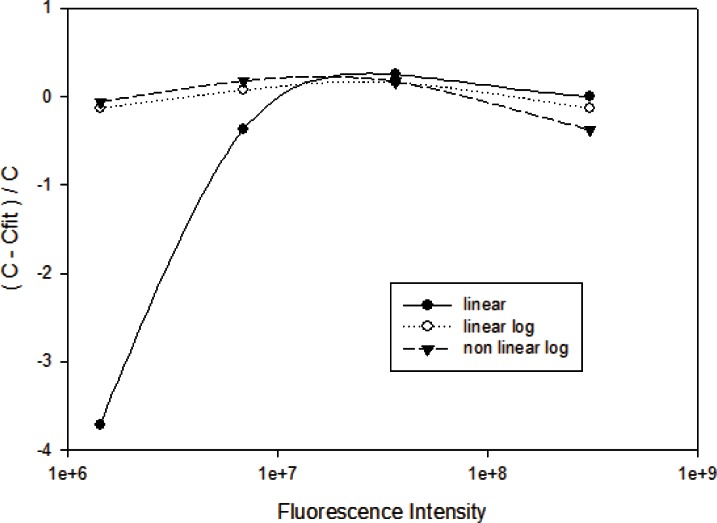
The deviations between the best fit and the intensity response of four solutions of APC with progressively larger concentrations of APC. The fit of the data with a line gives the largest deviations at smaller concentrations. Fits to logarithms of the data yield smaller deviations.

Figure 4 shows the results of fitting the data in Table 3 to obtain a calibration line. The fluorescence intensity is given on the horizontal axis, and the vertical axis shows the ratio of the deviations between the concentration data and the fitted values, and the concentration data. The solid circles give the results using Eq. (1) (linear fit to data), the open circles give the results using Eq. (5) (linear fit to log of data), and the solid triangles give the results using Eq. (3) (non linear fit to log of data). The linear fit gives large deviations at lower concentrations, while the fits to the log of the data yield similar deviations across the entire range of concentrations. Similar calibration curves were obtained for the FITC, Nile Red, and Coumarin [30] reference solutions (results not shown).

### 3.3 Fluorescence Intensity of the APC Microspheres

Three identical suspensions of APC microspheres were prepared according to the instructions supplied by the manufacturer. A PBS based buffer with 0.5 % (w/w) Bovine Serum Albumin (BSA) and 0.1 % (w/w) sodium azide was provided by the manufacturer of APC microspheres. Each suspension was transferred to a cuvette and fluorescence measurements were performed in a manner identical to those performed for the calibration solutions. An example of spectra is shown in Fig. 3c for the APC microspheres. Two measurements were performed in sequence to detect photodegradation. The fluorescence intensity was obtained in the same way as in the case of the reference solution. The average intensity value of the first fluorescence measurement of the three identical APC micro-sphere suspensions was (1.392 ± 0.011)*10^6^ and the average value of the second measurement was (1.356 ± 0.005)*10^6^. There appears to be some photodegradation, but the amount in terms of percentage is small compared to that observed for FITC microspheres (about 20 % reduction in fluorescence intensity in the subsequent measurement). In order to minimize effects of photodegradation, the first measurement was always used in the determination of the ERF value. Using the fluorescence intensity of the APC microspheres in the calibration line provided by the data in Table 3, we obtained the equivalent concentration of reference fluorophores shown in the second column in Table 4. The first column in Table 4 gives the type of fit used for the data in Table 3. The last row in Table 4 gives the equivalent concentration obtained by using the nearest calibration point to estimate the slope and neglecting the intercept (Solution 6 in Table 3). Use of the slope obtained from the nearest calibration point to estimate the concentration is the easiest method since it does not require any fitting of the data. The value obtained by the linear fit to data is significantly different from values by other fitting methods because of the larger fitting deviations at lower concentrations as shown in Fig. 4.

### 3.4 Concentration of APC Microsphere Calibrator

**Fig. 5:**
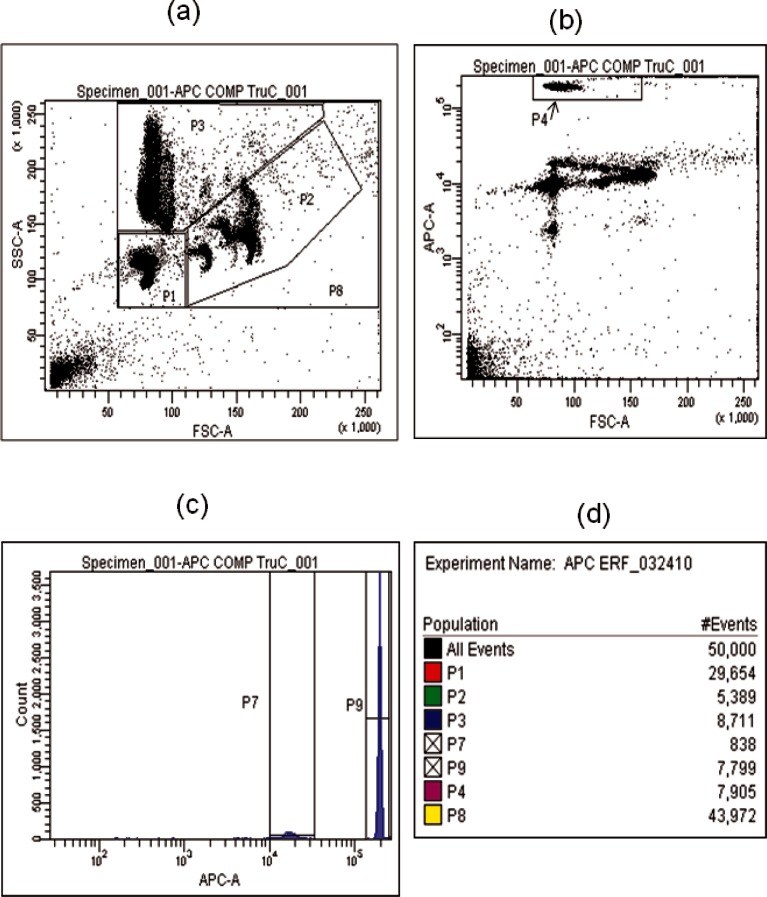
(a) A scatter plot of data from a sample containing TruCount beads and APC microspheres calibrator. The horizontal axis is the forward scattering signal and the vertical axis is the side scattering signal. Each dot corresponds to a single particle event recorded by the flow cytometer. The gate P8 encompasses almost all of the recorded particle events except the debris, and the regions P1, P2, and P3 represent different populations of the APC microspheres with P1 containing the major population. The TruCount bead events are contained in the P3 gate. (b) A scatter plot of the APC fluorescence signal on the vertical axis and the forward scattering signal on the horizontal axis. The gate P4 encloses all events associated with the TrueCount beads. (c) A histogram of the APC fluorescence signal for all events contained in gate P3 of Fig. 5a, (d) a compilation of the number of events associated with the gates in Figs. 5 a,b,c.

Figure 5 shows the results of a flow cytometer measurement on a sample containing the APC microsphere calibrator and the TruCount beads. The sample was made by re-suspending a tube of TruCount beads in 0.5 mL of the suspension of APC microsphere calibrator. (A portion of the same suspension of APC microspheres was used in the fluorescence measurements discussed above). When a microsphere passes through the laser beams of the flow cytometer, a total of six signals are detected and analyzed. These signals are the forward scattering, side scattering at 90°, and the four fluorescence channels (FITC, PE, APC and PB). The two scattering signals are used to classify the microspheres according to size and shape. The scatter plot in Fig. 5a with axes labeled SSC-A (side scatter channel) and FSC-A (forward scatter channel) shows several microsphere populations. The gate P8 encloses the scattering events associated with all microspheres and the counting beads in the sample. The gate P8 is further subdivided into regions associated with different particle populations. The gate P1 contains the major population of the APC microspheres. The gate P3 contains a minor part of the APC microspheres and the entire population of TruCount beads. The gate P2 contains another minor population of the APC microspheres. Fig. 5a does not give a graphical display of the relative intensity of the different populations; however it is simple matter to electronically count the number of dots in each gate. The result box (d) at the bottom of Fig. 5 gives the number of events associated with the P8 gate as 43972. The TruCount beads, contained in the P3 gate, have a large fluorescence signal in the APC fluorescence channel. The fluorescence signal can be used to identify and select the counting beads. Figure 5b shows a dot plot, labeled with axes APC-A and FSC-A, which was obtained from all events in Fig. 5a. The gate P4 in Fig. 5b gives the distribution of fluorescence signals associated with the TruCount beads. The events in P3 scatter plot are further displayed in a histogram in Fig. 5c with axis labels Count and APC-A. The gate P9 in the histogram gives the number of TruCount beads, the value of which is 7799. The gate P7 in the histogram gives the number of APC microspheres in the minor population contained in gate P3. The number of events in gates P4 and P9 for TruCount beads in Fig. 5 is within the measurement uncertainty, which was estimated from the spread of the distribution of events. Using 48918, provided by the manufacturer, for the given number of TruCount beads in the vial, Eq. (15) gives the concentration of APC microspheres as (453800 ± 36000) mL^−1^. The uncertainty associated with this measurement is about 8 % of the total value.

### 3.5 Assignment of ERF Values to APC Microsphere Calibrator

**Fig. 6:**
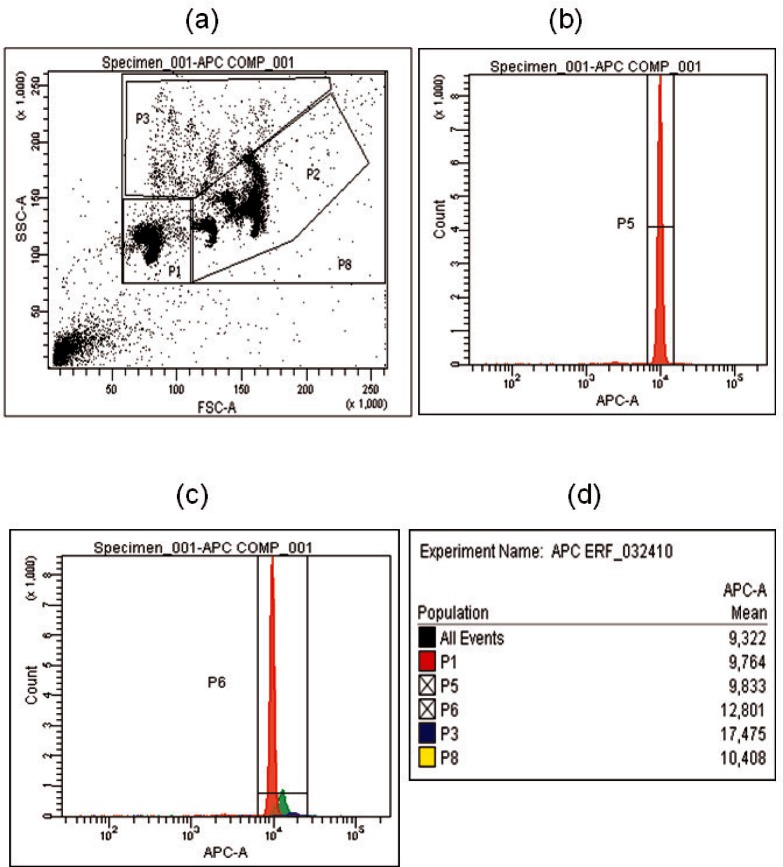
(a) A scatter plot of data from a suspension of APC microspheres calibrator. The horizontal axis is the forward scattering signal and the vertical axis is the side scattering signal. Each dot corresponds to a single APC microsphere recorded by the flow cytometer. (a) The gate P8 encompasses almost all of the recorded microsphere events except the debris, and the regions P1, P2, and P3 represent different populations of the microspheres. (b) A histogram of the fluorescence signal in the APC channel for events in gate P1. (c) A histogram of the fluorescence signal in the APC channel for events in gate P8. Note: not all microspheres are included in the gate P6. P6 only shows the majority of microspheres. (d) A compilation of the mean fluorescence intensity values associated with the gates in Fig 6 a,b,c.

In the previous sections, procedures were described for obtaining the microsphere concentration and the equivalent concentration of reference fluorophores for APC microspheres. Given these values, Eq. (17) was used to calculate the value of equivalent reference fluorophores (ERF). The second column of Table 5 gives the values of ERF obtained by using (453800 ± 36000) mL^−1^ for the microsphere concentration and the values of the equivalent fluorophore concentrations given in Table 4. The ERF values are for all microspheres. However, as mentioned in the discussion of Fig. 5, the APC calibration microspheres consist of a major population and two minor populations. The major population consists of the events in gate P1 in the SSC-FSC scatter plot in Fig. 5a. The minor populations consist of events in the P3 and P2 gates in the same scatter plot. Figure 6 is similar to Fig. 5, except that it represents a sample that contains only APC calibration microspheres without the TruCount beads as was the case in Fig. 5. Note that the density of events in gate P3 without the TruCount beads is much smaller in Fig. 6 than in Fig. 5 with TruCount beads. The fluorescence signals from the major and minor populations of the APC calibration microspheres are different. This was established by comparing the mean fluorescence channel of the events associated with the three gates in Fig. 6a. The resulting histogram is shown in Fig. 6c with mean fluorescence channels 9764 for P1, and 17475 for P3. Figure 6b shows the fluorescence histogram for events associated only with gate P1 in Fig. 6a. The gate P1 contains about 80 % of the events associated with the APC calibration microspheres and it is the gate that defines the APC microsphere event in the calibration of the flow cytometer and flow cytometry applications. Therefore, it is important to obtain an estimate of the ERF value associated with this major microsphere population in the gate P1. This can be accomplished by multiplying the ERF values given in column 2 of Table 5 by the ratio of mean fluorescence signal of events in gate P1 and P8 in Fig. 6a, or specifically by P1/P8 = 9764/10408 = 0.938(Eq. (18)). The ERF values of the major population of the APC calibration microspheres are given in the third column of Table 5. The uncertainties in the ERF values are about 8 % of the actual value and were obtained from the uncertainties of the values of *C_equivalent_* and *C_microspheres_*. Additional systematic uncertainties are introduced by the choice of the calibration algorithm.

## 4. Conclusion

The detailed procedure outlined for the APC fluorescence channel was also performed for three other fluorescence channels: FITC, PE, and Pacific Blue (PB). The resulting values of ERF for the major component of each microsphere calibrator, determined using the nearest calibration point method, are shown in the second column of Table 6. The third column gives the name of the reference fluorophore. The four populations of microspheres would give a single point measurement in the four fluorescence channels. The calibration could be extended to multiple points by developing software controls to change the photomultiplier voltages and collect data at each value of the voltage. Alternately, a calibration could be obtained with multiple populations of microspheres, each with a different fluorescence intensity and a different ERF assignment. Once the linear ERF scale is established, another reference standard will be used to change the ERF scale to a scale giving the antibodies bound to the cell. The nature of this second reference standard is the topic of a subsequent paper.

## Figures and Tables

**Table 1:** Weightings of the APC stock solution, all values in grams

Tube	tube + APC	tube + APC+PBS
7.87937	7.97237	17.89261
7.87937	7.97249	17.89320
7.87958	7.97345	17.89361

**Table 2:** Concentrations of reference solutions of APC

Solution name	Concentration, mol/L	Uncertainty, Mol/L
Solution 6	2.64e-11	1.4e-12
Solution 5	1.32e-10	6.7e-12
Solution 4	6.56e-10	3.4e-11
Solution 3	3.29e-9	1.7e-10

**Table 3:** Spectrometer calibration with reference solutions of APC

Solution name	Concentration, mol/L	Uncertainty, mol/L	Fluorescence Signal, AU
Solution 6	2.64e-11	1.4e-12	1.43e6
Solution 5	1.32e-10	6.7e-12	6.85e6
Solution 4	6.56e-10	3.4e-11	3.65e7
Solution 3	3.29e-9	1.7e-10	3.07e8

**Table 4:** Equivalent concentration of reference fluorophore APC*

Type of calibration	Value, mol/L
Linear fit to data	1.241e-10
Linear fit to log of data	2.917e-11
Non linear fit to log of data	2.749e-11
Nearest calibration point	2.578e-11

* The uncertainties are about 8 % of the actual values

**Table 5:** Assigned values of equivalent reference fluorophore (ERF) APC calibration microspheres

Type of calibration	All microspheres, ERF	ERF of the major population of microspheres*
Linear fit to data	16470	15400
Linear fit to log of data	38720	36300
Non linear fit to log of data	36490	34200
Nearest calibration point	34220	32100

* uncertainties are about 12 % of the values

**Table 6:** Assigned values of equivalent reference fluorophore

Microsphere	ERF	Reference fluorophore
FITC	7.74e04 ± 9.1e03	FITC (SRM)
PE	7.94e05 ± 9.1e04	Nile Red
APC	3.21e04 ± 4.0e03	APC
Pacific Blue (PB)	1.59e06 ± 2.0e05	Coumarin 30

## Footnotes

1 Certain commercial equipment, instruments, and materials are identified in this paper in order to specify the experimental procedure as completely as possible. In no case does this identification imply a recommendation or endorsement by the National Institute of Standards and Technology, nor does it imply that the equipment, instruments, or materials identified are necessarily the best available for the purpose.
